# Prevalence of anal symptoms in general practice: a prospective study

**DOI:** 10.1186/s12875-017-0649-6

**Published:** 2017-08-03

**Authors:** Géraldine Tournu, Laurent Abramowitz, Camille Couffignal, Frédéric Juguet, Agnès Sénéjoux, Stéphane Berger, Anne-Laure Wiart, Marc Bernard, Françoise Provost, Hélène Pillant-Le Moult, Dominique Bouchard, Jean-Pierre Aubert, for the GREP study group, for the MG-PREVAPROCT study group

**Affiliations:** 1 General practice department, University Paris Diderot, F-75018 Paris, France; 2 AP-HP and GREP; Gastroenterology and proctology unit, Bichat University hospital, Paris, France; 3 INSERM, IAME, UMR 1137, F-75018 Paris, France; 4 University Paris Diderot, IAME, UMR 1137, Sorbonne Paris Cité, F-75018 Paris, France; 5 AP-HP, Bichat hospital, Biostatistics unit, F-75018 Paris, France; 6 Tivoli Ducos Clinic, Bordeaux, France; 7 Proctology unit, Private hospital, Saint Grégoire, France; 8 Montigny les Cormeilles, France; 9 Bordeaux, France; 10 Cadaujac, France; 11 Rennes, France; 12 Blomet Clinic, Paris, France; 13 Proctology unit, Bagatelle hospital, Talence, France; 14 Sorbonne Paris Cité, General practice department, University Paris Diderot, F-75018 Paris, France ; REMES, F-75018 Paris, France

**Keywords:** General practice, Proctology, Anal, Examination, Epidemiology, Haemorrhoids

## Abstract

**Background:**

Anal disorders are largely underestimated in general practice. Studies have shown patients conceal anal symptoms leading to late diagnosis and treatment. Management by general practitioners is poorly described. The aim of this study is to assess the prevalence of anal symptoms and their management in general practice.

**Methods:**

In this prospective, observational, national study set in France, all adult patients consulting their general practitioner during 2 days of consultation were included. Anal symptoms, whether spontaneously revealed or not, were systematically collected and assessed. For symptomatic patients, the obstacles to anal examination were evaluated. The general practitioner’s diagnosis was collected and a proctologist visit was systematically proposed in case of anal symptoms. If the proctologist was consulted, his or her diagnosis was collected.

**Results:**

From October 2014 to April 2015*,* 1061 patients were included by 57 general practitioners*.* The prevalence of anal symptoms was 15.6% (95% CI: 14–18). However, 85% of these patients did not spontaneously share their symptoms with their doctors, despite a discomfort rating of 3 out of 10 (range 1–5). Although 65% of patients agreed to an anal examination, it was not proposed in 45% of cases with anal symptoms. Performing the examination was associated with a significantly higher diagnosis rate of 76% versus 20% (*p* < 0.001). Proctologist and general practitioner diagnoses were consistent in 14 out of 17 cases.

**Conclusions:**

Patients’ concealed anal symptoms are significant in general practice despite the impact on quality of life. Anal examination is seldom done. Improved training of general practitioners is required to break the taboo.

## Background

General practitioners (GP) should be first in line to manage anal disorders, given their high prevalence in the general population and their simple medical treatment. However, epidemiological research concerning general practice is poor and difficult to obtain because of patients’ reluctance to discuss symptoms and to agree to anal examination. Existing studies have evaluated anal symptoms only by questioning patients, without any anal examination. Therefore results of the prevalence of anal disorders vary, from 20% to 40.5% [1–3]. In 2014, Abramowitz et al. evaluated the prevalence of anal symptoms in general practice. Among 1079 patients who consulted their GP in France, 2% consulted spontaneously for an anal symptom. However, after systematic questioning of all patients, the prevalence was in fact 14.2% [4]. Patients do not freely discuss their symptoms, despite an effect on quality of life [5]. Furthermore, anal examination is seldom done by doctors [4], even though it is considered important [6]. A 1990 study assessed the practice of family doctors in England. Reasons for not performing a rectal examination included patients’ reluctance, lack of time or repetition of the examination by a proctologist [7].

To pursue the research by Abramowitz et al. [4], we conducted this study to assess the prevalence of anal symptoms, this time by systematic questioning and clinical examination, and to evaluate their management in general practice, by assessing the diagnostic approach, the clinical examination proposal rate, the diagnosis rate and the frequency of referral to a proctologist.

## Methods

### GP recruitment

This observational prospective study was conducted in 5 cities in France: Beausoleil, Bordeaux, Nîmes, Paris and Rennes. Eight referent proctologists were recruited by the French group for proctological research (GREP). Secondly, each proctologist provided a list of his or her usual contact GPs, who were recruited: two hundred and fifty one GPs were contacted by telephone. Eight centres were organised around the 8 proctologists and their contact GPs (1 centre in Beausoleil, 3 centres in Bordeaux, 1 centre in Nîmes, 2 centres in Paris and 1 centre in Rennes). Prior to the study, an optional 2 h medical lecture on anal disorders was proposed to all GPs.

### Patient recruitment

All patients were included over 1 to 2 days of consultation. Each GP was first given the option of choosing 1 day within a predefined week for inclusion, but due to lack of GP participation, an additional day was proposed. GPs proposed participation to all consecutive adult patients. Home visit patients were excluded. Data on the reason for refusal to participate was also documented.

### Data collection

The presence of anal symptoms, at the time or over the past month, was assessed using a self-administered survey, completed in the waiting room by the patient just before consultation. Patients could choose among a list of symptoms: bleeding, anal pruritus, anal pain, anal swelling, anal discharge and uncontrolled anal leakage. They specified if the anal symptom was the reason for consultation and assessed the level of discomfort and pain on a 0 to 10 numeric rating scale. For each patient included, GPs completed an electronic Case Report Form during consultation. They identified the presence of constipation and diarrhoea. GPs then proposed an anal examination to all symptomatic patients. If they did not propose the examination, the reason of refusal was documented.. Proctologic diagnoses and prescription were also recorded. Additionally, GPs estimated the time dedicated to the proctological issue during the consultation, from patient questioning to prescription.

### Proctologist consultation

Patients with anal symptoms were offered a consultation with a proctologist, which would take place within 3 days after the GP visit. If the patient refused, the cause was documented by the GP during consultation.

### Statistical methods

Statistical analysis was made for all patients with complete data. Patients with missing data from the self-administered survey were excluded. Continuous variables were expressed as median and range and categorical variables as counts and percentages. To compare the distribution of quantitative variables, the Wilcoxon rank sum test was used. To compare the distribution of qualitative variables, the chi-square test was used.

The concordance rate between the GP and the proctologist diagnoses were measured by the Kappa coefficient. All analyses were performed using SAS software (SAS Institute, Cary, NC). Statistical significance level was defined by *p* < 0.05.

A sample size of 1920 participants would allow an accuracy of 3% for an expected prevalence of 14% as shown in the previous study by Abramowitz et al. [4]. For each of the 8 referent proctologists, 15 GPs were to be contacted. Considering an average of 20 patients seen each day in each GP office in France and an estimated refusal rate of 20%, this would lead to an estimated inclusion of 1920 participants. One centre in Paris was excluded before the beginning of the study due to the lack of GP participation.

## Results

### Population included

**Fig. 1:**
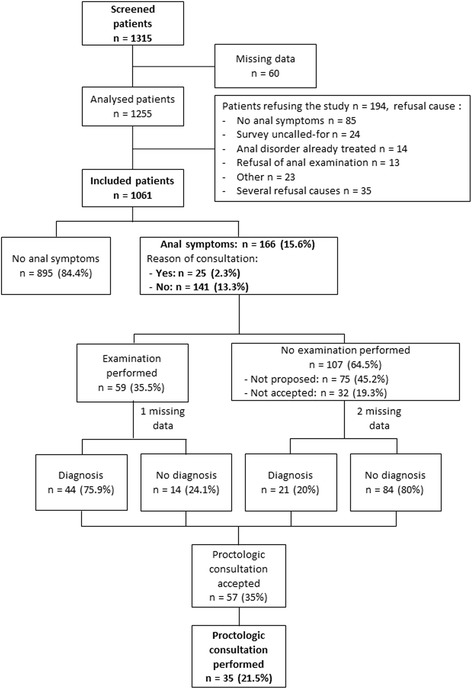
Patient flow-chart

Among 251 doctors contacted, 80 accepted the study and 59 included at least 1 patient. The two main reasons for doctors’ refusal to participate were lack of time or interest in the research. Doctors’ average age was 50 years (range 42–57) and 52.6% were male. Two doctors who did not follow the research protocol during patient inclusion were excluded. Assessment took place from October 2014 to April 2015, and 1315 patients were screened by the 57 doctors. The median number of patients assessed by each doctor was 19 (range 15–29). Among the 1315 screened patients, 60 patients were excluded for missing age or sex, and 1061 (84.5%) accepted to participate in the study. The median age was 52 years (range 37–67), 414 were males (39%). Causes for refusal to participate are listed in Fig. 1.

### Prevalence of anal symptoms

After targeted questioning, anal symptoms were found for 166 patients (15.6%; 95% CI: 14–18), of which only 25 (2.3%; median age: 43 years, range 32–54) declared it was the reason for consultation. One hundred forty-one patients (13.3%; median age: 49 years, range 38–61) had an anal symptom though this was not the reason for consultation (Table 1). No difference in distribution of anal symptoms was found between centres.

**Table 1:** Prevalence of anal symptoms and diagnosis approach

Anal symptom	Total anal symptoms (*n* = 166)	Reason for consultation (*n* = 25)	Revealed by questioning (*n* = 141)	*p* value^a^
Anal symptom				
Bleeding	77 (46.4%)	13 (52%)	64 (45.4%)	0.66
Anal pruritus	74 (44.6%)	11 (44%)	63 (44.7%)	1
Pain	57 (34.3%)	17 (68%)	40 (28.4%)	<0.001
Anal swelling	45 (27.1%)	12 (48%)	33 (23.4%)	0.01
Anal discharge	17 (10.2%)	1 (4%)	16 (11.3%)	0.47
Uncontrolled anal leakage	15 (9%)	3 (12%)	12 (8.5%)	0.70

^a^chi-square test *p*-value

Patients spontaneously consulting for an anal complaint in comparison with patients who revealed the symptoms after questioning

### Anal symptom and constipation or diarrhoea

GPs assessed the presence of constipation or diarrhoea for patients with anal symptoms (Table 2). Constipation and a combination of diarrhoea and constipation were associated with the presence of an anal symptom. Among the 127 patients with constipation, 50 (39.4%) had anal symptoms. Likewise, among the 44 patients with a combination of diarrhoea and constipation, 22 (50%) had anal symptoms.

**Table 2:** Prevalence of diarrhoea and constipation, depending on the presence of an anal symptom (*n* = 1061)

Intestinal transit disorders (*n* = 249)	Patients with anal symptoms (*n* = 166)	Patients without anal symptoms (*n* = 895)	*p*-value^a^
Diarrhoea (*n* = 78)	17 (10.2%)	61 (6.8%)	0.12
Constipation (*n* = 127)	50 (30.1%)	77 (8.6%)	< 0.001
Both (*n* = 44)	22 (13.3%)	22 (2.5%)	< 0.001

^a^chi-square test *p*-value

### Proctological diagnoses

A diagnosis was made for 65 patients out of 165 (39.9%), and there was no diagnosis for 98 patients (60.1%). Diagnoses included: 42 haemorrhoids (25.8%), 10 anal fissures (6.1%), 3 dermatologic disorders (1.8%), 2 condyloma (1.2%), 2 anal abscesses, 2 marisca, 2 idiopathic pruritus, 1 mycosis, 1 lichen, 1 anal erythema, 1 anal excoriation, 1 anal incontinence after surgery and 2 unidentified disorders. Five patients had more than one diagnosis (2 patients were diagnosed with haemorrhoids and an anal fissure, 1 patient with haemorrhoids and a mycosis, 1 patient with an abscess and a dermatologic disorder and 1 patient with an anal fissure and a condyloma).

**Fig. 2:**
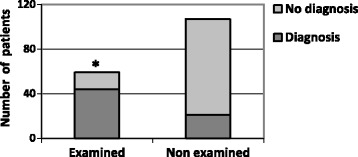
Proportion of diagnoses depending on clinical examination

Performing an anal examination was associated with a higher diagnosis rate of 76% versus 20% (*p* < 0.001) (Fig. 2). Not performing an anal examination in case of anal symptoms resulted in an increased risk of non-diagnosis with a relative risk (RR) of 3.5; 95% CI: 2.1–5.6.

### Pain and discomfort

Pain and discomfort induced by anal symptoms were respectively 2 out of 10 (range 0–5) and 3 out of 10 (range 2–6) for all symptomatic patients. Patients with a spontaneous anal complaint rated the pain 5 out of 10 (range 2–7) versus 1 out of 10 (range 0–4) for patients who did not consult for the symptom (*p* = 0.001). Likewise, discomfort was more important among patients with a spontaneous anal complaint, 5 out of 10 (range 3–8) versus 3 out of 10 (range 1–5) (*p* = 0.006) for patients who did not consult for the symptom.

**Fig. 3:**
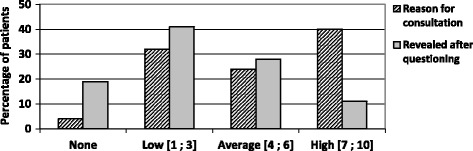
Level of discomfort due to the anal symptom, when it was spontaneously declared or revealed after questioning

Discomfort was considered average or high in 43% of cases for patients with a spontaneous anal complaint and for 40% of cases for patients who did not consult for the symptom (Fig. 3).

### Anal examination in general practice

GPs did not propose any anal examination for 75 out of 165 (45.2%) patients with anal symptoms, for different reasons (Table 3). None of the GPs declared being constrained by the anal examination. Among the 59 patients who accepted the examination, all underwent an anal margin examination, but digital rectal examination was not done for 24 patients (40.7%).

**Table 3:** Anal examination in general practice

Reasons GPs did not propose an anal examination	Patients (*n* = 75)
Another predominant cause of consultation	*n* = 24 (32%)
Lack of time	*n* = 21 (28%)
Patient already under treatment	*n* = 19 (25%)
Fear of embarrassing the patient	*n* = 4 (5%)
Patient directly addressed the proctologist	*n* = 4 (5%)
Other	*n* = 9 (1%)
Reasons GPs did not perform a digital rectal examination	Patients (*n* = 24)
Fear of causing pain	*n* = 10 (42%)
Absence of indication	*n* = 5 (21%)
Patient’s reluctance	*n* = 3 (12%)
Embarrassment	*n* = 2 (8%)
Lack of knowledge in proctology	*n* = 2 (8%)
Lack of time	*n* = 2 (8%)
Other	*n* = 5 (21%)
Reasons patients refused the anal examination	Patients (*n* = 32)
Discomfort	*n* = 11 (34%)
Anal symptoms considered of minor importance	*n* = 11 (34%)
Examination already performed for the symptom	*n* = 8 (25%)
Knowing the examination would be repeated by the specialist	*n* = 7 (22%)
Lack of time	*n* = 4 (12%)
Other	*n* = 2 (6%)

The anal examination was refused by 32 patients (35.2%) for various reasons (Table 3). None declared a fear of a painful examination.

For the 166 patients with anal symptoms, GPs estimated dedicating 6 min (range 5–10) to the proctological issue, from interrogation to prescription. When the patient did not undergo an anal examination, estimated time was 5 min (range 3–8). Comparatively, when the GP performed an examination, estimated time was 10 min (range 8.5–15).

### Treatments

Haemorrhoids was the most frequent diagnosis. For those patients with this diagnosis, ointments were then most often prescribed (69%), followed by suppositories (45.2%), phlebotonic drugs (31%), laxatives (28.6%), oral anti-inflammatory drugs (21.4%) and oral analgesics (14.3%). Phlebotonic, also known as venoactive, drugs increase venous tone leading to an increase in capillary resistance, improvements in lymphatic flow and reduction in oedema.

### Proctologist’s follow-up consultation

Among the 166 patients with anal symptoms, fifty-seven (34.9% on 163 with complete data) accepted a consultation with the referent proctologist. Thirty-five (21.2%) patients were seen in consultation. Among them, 17 had a diagnosis from the GP, of which 14 were in concordance with the proctologist’s diagnosis (Kappa = 0.66; 95% CI: 0.27–0.91).

### Medical lecture

Among the 57 participating GPs, 40% (*n* = 23) attended the lecture before screening. The lecture did not have any impact on their practice. The rate of anal symptoms found by patient questioning was no different. Furthermore, GPs who attended the lecture did not perform more anal examinations.

## Discussion

### Summary

For this study we assessed the diagnostic approach, the clinical examination proposal rate, the diagnosis rate and the frequency of referral to a proctologist.

In our cohort of 1061 patients, 2.3% spontaneously consulted their GP for an anal symptom. However, after questioning all patients, the prevalence of anal symptoms was 15.6% (95% CI: 14–18). This not only reveals the high prevalence of anal symptoms in general practice but also highlights that they are under-reported by patients and underestimated by practitioners.

Furthermore, half the patients presenting a combination of diarrhoea and constipation had an anal symptom.

While the main diagnoses were haemorrhoids (25.8%) and anal fissures (6.1%), 60.1% of patients had no diagnosis. Only 54.8% of symptomatic patients were proposed an anal examination by their GP. Yet, our results show that performing an examination was associated with a higher diagnosis rate, of 76% versus 20% (*p* < 0.001). When the examination was not done, there was an increased risk of non-diagnosis. This highlights the importance of the examination in the diagnosis approach. GPs estimated devoting 5 min to the proctological issue when the examination was not done and 10 min when it was done.

We studied the management of anal disorders by GPs. Although anal examination was not proposed for 45.2% of patients, none of the GPs declared being constrained by it. They complained of lack of time, especially because of another reason of consultation. In other cases, they were afraid of embarrassing the patient or they directly addressed the patient to the proctologist.

Concerning patients, anal examination was accepted by the majority (64.8%) but still refused by a third. The principal reason was discomfort, followed by the fact that the anal symptoms were considered of minor importance. In other cases, patients refused, knowing the examination would be repeated by a specialist or because they were in a hurry. One of the factors influencing the patient’s decision could be the doctor’s confidence and motivation.

Finally, concerning treatments, first line treatment in haemorrhoids and anal fissures involves treating constipation and dyschesia [8–10]. Yet in our study, for haemorrhoids, laxatives were not prescribed in 28.6% of cases and no treatment was given in 14.3% of cases. Likewise, for anal fissures, no treatment was given in 20% of cases and only half of patients were prescribed laxatives.

### Strengths and limitations

This is the first study to have prospectively assessed the management of anal symptoms in general practice, by describing the patient’s path from their GP consultation to the proctologist’s follow-up visit. This observational study presents limits. First, due to low GP participation, it was necessary to increase inclusion per GP, by setting up an additional day of inclusion during consultation. This could have led to a recruitment bias, as the volunteering GPs could have been sensitized to anal diseases and their medical practice does not necessarily reflect the general practice. Inclusion rate was lower than intended, diminishing the study’s strength. Yet our data show no difference between the 7 centres. Distribution was similar in different French cities, hence representing French medical practices. Secondly, anal symptoms were sought up to 1 month preceding consultation. Therefore patients could have no more symptoms at the time of the study, leading to a low rate of examination and a high rate of non-diagnosis in our study (60.1%). Finally, the referent proctologist saw few patients, meaning the GPs diagnoses were confirmed only for a few cases.

### Comparison with existing literature

A study by Nelson et al. found a prevalence of anal symptoms of 20% in the general population, using a telephone survey with 102 adults in the Joliet, Illinois area [1]. Abramowitz et al. found similar results to our work: 14.2% of anal symptoms [4] in France but among a different population of GPs and in different areas of the country. The concealment of symptoms was already reported by Siproudhis et al. with 58.3% of patients suffering from proctological disorders who never consulted their GP [2]. In our study, 85% of patients revealed their symptoms only after questioning.

Concerning anal symptoms, our data was similar to those of a French study by Pigot et al. where 161 doctors recruited 831 patients consulting for a proctological complaint. Symptoms recorded using an information sheet were: pain (48%), bleeding (37%), anal swelling (26%) and anal pruritis (24%) [3]. In this study, the subjective impact of anal symptoms (none, little, important, enormous) on quality of life was assessed. It was considered important or enormous for 44% of patients who consulted for that symptom. In our work, on a numeric rating scale from 0 to 10, discomfort was considered average or high in 43% of cases. Our study is the first to evaluate the impact on patients who had not spontaneously revealed their symptoms. For these patients, discomfort was average or high in 40% of cases.

Regarding doctors’ diagnosis approach, our survey confirms the low rate of anal examination, especially rectal examination. In a study by Springall and Todd, among 305 patients with anal or digestive symptoms addressed by their GP to the specialist, 31% were not examined and only 48% underwent a rectal examination by the GP [6], similar to our data where an examination was not proposed in 45.2% of cases.

### Implications for practice

Patients don’t spontaneously reveal anal symptoms, despite affecting quality of life. Patient discomfort towards anal examination could be a restraint. A systematic screening for anal disorders is already recommended for HIV (Human Immunodeficiency Virus) patients [11] and is common practice for pregnancy and postpartum patients [12]. Screening should also be systematic in case of constipation and/or diarrhoea, given the high rate of anal symptoms in these populations.

In parallel, informing patients about the frequency of anal disorders could help encourage patients to spontaneously share their symptoms, to avoid inadequate self-medication or aggravation of certain disorders. Similarly, medical training for students and practitioners could sensitize the medical profession, especially the importance of examination, which is seldom done and could lead to absence or error in diagnosis. Regarding anal fissures, late diagnosis can lead to chronic or infected fissures, which could put patients at risk of faecal incontinence due to surgery. As for haemorrhoids, simple medical or instrumental treatment is often sufficient, but late diagnosis could also lead to complications due to necessary surgery. Finally, there is the risk of overlooking an anal or rectal cancer.

## Conclusion

2.3% of patients consult GPs spontaneously for an anal complaint. However, after targeted questioning of patients, 15.6% (95% CI = 14 to 18) of adult patients have an anal symptom. Despite the impact on quality of life, leading to discomfort and pain, these symptoms are seldom declared.

In 60.1% of cases, GPs do not diagnose for reported anal symptoms. It is of note that performing an anal examination is associated with a 3.8 times higher diagnosis rate. Yet examination is insufficiently proposed by GPs. When proposed, it is accepted by patients two thirds of the time. To break the taboo and improve management, better information concerning anal symptoms could be provided to both patients and practitioners.

## Abbreviations

GP: General practitioner
HIV: Human Immundeficiency Virus
