# Correction to: Prevalence of anal symptoms in general practice: a prospective study

**DOI:** 10.1186/s12875-019-0905-z

**Published:** 2019-01-17

**Authors:** Géraldine Tournu, Laurent Abramowitz, Camille Couffignal, Frédéric Juguet, Agnès Sénéjoux, Stéphane Berger, Anne-Laure Wiart, Marc Bernard, Françoise Provost, Hélène Pillant-Le Moult, Dominique Bouchard, Jean-Pierre Aubert, for the GREP study group, Cécile Laclotte, Dominique De Faucal, Thierry Higuero, Guillaume Bonnaud, Vincent De Parades, Véronique Vitton, for the MG-PREVAPROCT study group, Cédric Laouénan, Sandrine Calonne, Sonia Corral, Philippe Uge, Thomas Tarjus, Raymond Wakim, Thierry Dubon, Pierre Fenouillet, Gregory Lovato, Alain Lovato, Valérie Berthelot-Calmels, Philippe Denoyelle, Gregory Moisset, Anne Youssefian, Valérie Douillard, Michel Geffrault, Marie Pierre Bouchara, Mathieu Petit, Bertrand Leblanc, Claudine Brune Ropert, Emmanuel Fournier, Cécile Andreu, Isabel Ferraris, David Ferraris, Nora Gauffier, Ismaël Nureni Banafunzi, Claire Salanova, Michael Boyadjian, Joelle Desaize, Catherine Dubertret, Cornélis Floor, Catherine Majerholc, Ewa Rudnicka, Hélène Vitou, Hélène Carrez-Louys, Frédéric Dumas, Monique Horwitz-Guérin, Marina Lavigne, Valérie Lubert, Eric Penichou, Julien Pouyanne, Sophie Sauvage-Rigal, Igor Sozonoff, Sandrine Baux, Marianne Guern, Bruno Calmels, Jamila Jamil, Catherine Chaumie, Guy Millou, Bernard Foinant, Alain Frangeul, Pascal Roger, Garry Magendie, Didier Marion, Jean-François Toledo

**Affiliations:** 1 General Practice Department, University Paris Diderot, F-75018 Paris, France; 2 AP-HP and GREP, Gastroenterology and Proctology Unit, Bichat University hospital, Paris, France; 3 INSERM, IAME, UMR 1137, F-75018 Paris, France; 4 University Paris Diderot, IAME, UMR 1137, Sorbonne Paris Cité, F-75018 Paris, France; 5 AP-HP, Bichat Hospital, Biostatistics Unit, F-75018 Paris, France; 6 Tivoli Ducos Clinic, Bordeaux, France; 7 Proctology Unit, Private hospital, Saint Grégoire, France; 8 Montigny les Cormeilles, France; 9 Bordeaux, France; 10 Cadaujac, France; 11 Rennes, France; 12 Blomet Clinic, Paris, France; 13 Proctology Unit, Bagatelle Hospital, Talence, France; 14 Sorbonne Paris Cité, General practice department, University Paris Diderot, F-75018 Paris, France; REMES, F-75018 Paris, France

**Correction to: BMC Fam Pract**

**https://doi.org/10.1186/s12875-017-0649-6**

Following publication of the original article [1], the authors reported an error to one of the ‘study groups’ in the authorship section. The collaborators of the study groups were not listed properly during typesetting. The members of the study groups are included here. The publisher would like to apologize for this error.
